# Coexistence of bilateral macular edema and pale optic disc in the patient with Cohen syndrome

**DOI:** 10.1515/med-2021-0208

**Published:** 2021-01-19

**Authors:** Klaudia Rakusiewicz, Krystyna Kanigowska, Wojciech Hautz, Dorota Wicher, Marlena Młynek, Marta Wyszyńska, Anna Rogowska, Joanna Jędrzejczak-Młodziejewska, Małgorzata Danowska, Agnieszka Czeszyk

**Affiliations:** Department of Pediatric Ophthalmology, Children’s Memorial Health Institute, Warsaw, Poland; Department of Medical Genetics, Children’s Memorial Health Institute, Warsaw, Poland

**Keywords:** Cohen syndrome, macular edema, pale optic disc, facial dysmorphism, CGH test

## Abstract

**Background:**

Cohen syndrome (Q87.8;ORPHA:193; OMIM#216550) is an autosomal recessive inherited genetic disorder caused by mutation in the *VPS13B/COH1* gene. It is characterized by variable clinical symptoms such as deformity of the head, face, hands and feet, eye abnormalities, abdominal obesity, neutropenia and nonprogressive intellectual disability. The typical lesions in the eyeball in Cohen syndrome include high myopia, retinal dystrophy, strabismus, maculopathy and lens subluxation. The present study describes the coexistence of bilateral macular edema with pale optic disc in a patient with a homozygous deletion in the *VPS13B/COH1* gene.

**Material and methods:**

A 6-year-old Caucasian girl with facial dysmorphism, microcephaly, prominent upper incisors, narrow hands with slender fingers, congenital heart defect and ophthalmic symptoms was subjected to genetic testing. The genetic evaluation revealed a homozygous deletion on the long arm of chromosome 8 encompassing 20–25 exons of the *VPS13* gene, as confirmed by Cohen syndrome. She underwent a full ophthalmological examination with the assessment of slit lamp examination of anterior segment and fundoscopy, refraction error, biometry, central corneal thickness and additionally electroretinography, optical coherence tomography and fundus photography.

**Results:**

In the ophthalmologic examination, the girl had bilateral astigmatism accompanied by myopia and a marked reduction in central corneal thickness. Fundus examination showed pale optic nerve discs and “salt and pepper” retinopathy. Bilateral cystic macular edema was revealed in handheld optical coherence tomography. Electroretinography showed a reduced response amplitude of cones and rods.

**Conclusion:**

In a patient with high myopia, macular edema, pale optic disc and facial dysmorphism, Cohen syndrome should be considered in the differential diagnosis. The severity of individual clinical features in patients with Cohen syndrome varies. It can be assumed that the type of mutation affects the occurrence and severity of individual symptoms.

## 1 Introduction

Cohen syndrome (Q87.8;ORPHA:193; OMIM#216550) is a rare genetic disorder that is inherited in an autosomal recessive manner [1,2,3,4,5,6]. The diagnosis is mainly based on the clinical picture, but no unequivocal diagnostic criteria have been established so far [5,7]. A genetic test that reveals mutations in the *VPS13B* gene, also known as *COH1*, confirms the diagnosis of Cohen syndrome [1,2,3,4,5]. The gene is located on the long arm of chromosome 8q22.2, and the gene protein product plays a role in protein sorting, vesicle-mediated protein transport, glycosylation and lysosomal function [1,3]. As a consequence, mutation in the gene leads to the production of defective, faulty, inefficient proteins. Depending on the function of a particular protein, it affects many systems and organs.

In 1973, Cohen et al. [2] described the syndrome by presenting three patients with similar, representative facial features with concomitant obesity, diminished muscle tone (hypotonia) and nonprogressive intellectual disability.

In 1987, Norio et al. [8] examined six patients in the Finnish population with similar clinical manifestations and additionally observed ocular symptoms such as myopia and retinal dystrophy.

The frequency of the disorder in the general population is unknown. Cohen syndrome occurs more frequently in the Finnish people and in the Amish families [2].

Based on the literature, it appears that the intensity of typical clinical features in patients varies greatly [1,5]. Identification of new mutations in *VPS13B* also presents extensive heterogeneity, which may explain clinical variability in Cohen syndrome [4]. However, there is no observed consistent correlation between specific mutations and the severity of specific clinical features [4,5].

Cohen syndrome is characterized by low birth weight, delay in reaching normal milestones in infancy and diminished muscle tone [1,2,4,5]. The affected individuals usually have a distinct appearance such as short stature, small, narrow hands and feet, microcephaly, a prominent nasal bridge, almond-shaped palpebral fissures, long eyelashes, thick eyebrows and hair. Most children with Cohen syndrome are described as sociable, open minded with a cheerful disposition [1,2,4,5].Typical features also include mental impairment, abdominal obesity (appearing in after mid-childhood) and intermittent chronic neutropenia associated with compromised immunity [1,2,4].

Abnormalities of the eyes described in patients with Cohen syndrome include progressive myopia and “salt and pepper” retinopathy [1,2,4]. Other ophthalmic symptoms consist of strabismus, iris coloboma, choroidal coloboma, posterior subcapsular cataract, astigmatism, microcornea, microphthalmia, maculopathy, ptosis, optic atrophy, exophthalmos and lens subluxation [1,2]. Although the symptoms of visual disturbances are serious, they usually do not lead to blindness. According to some authors, optimal vision is preserved up to the fourth decade of life [1,9].

## 2 Case study

**Figure 1:**
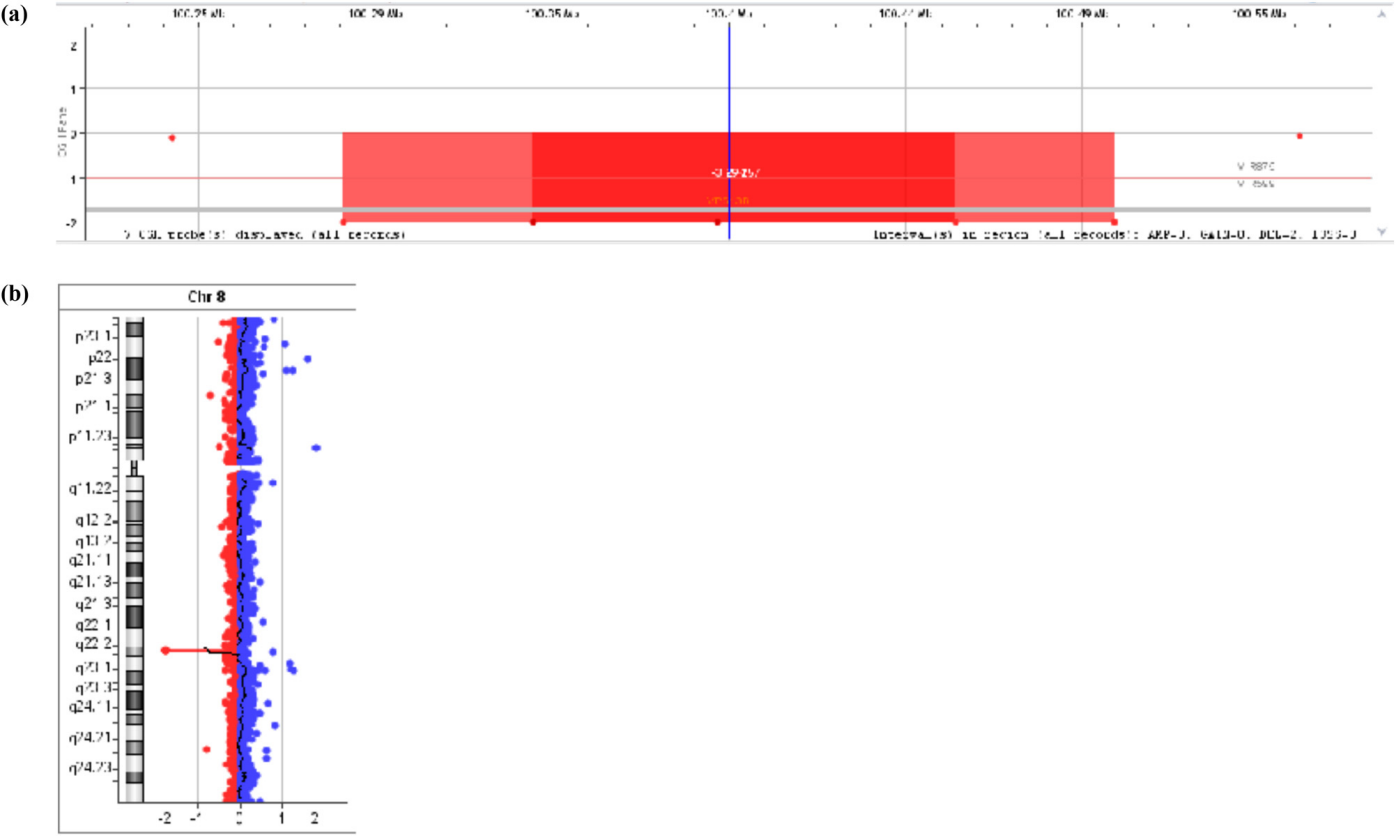
(a) and (b) Results of a CGH analysis pointing a partial loss of both copies of genetic material at the *VPS13B* gene: 8q22.2(100290888_100508951)x0 (Agilent Technologies SurePrint G3 ISCA V2 CGH 8x60K [hg19]).

In this case, a 6-year-old Caucasian girl is presented. She has been diagnosed with Cohen syndrome and is under multi-specialized care at the Children’s Memorial Health Institute. Informed consent has been obtained from parents of the patient presented in this study. In the array CGH testing, the presence of 218 Kb homozygous deletion in the long arm of chromosome 8 (region q22.2) was detected in the girl [Figure 1]. The identified deletion encompassed the 20–25 exons of the OMIM *VPS13B* gene, so these findings confirmed the diagnosis of Cohen syndrome.

**Figure 2:**
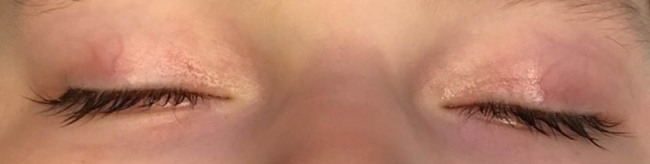
The girl with Cohen syndrome – note thick hair and eyebrow, abnormalities of the palpebral fissures (downslanting and almond-shaped palpebral fissures).

**Figure 3:**
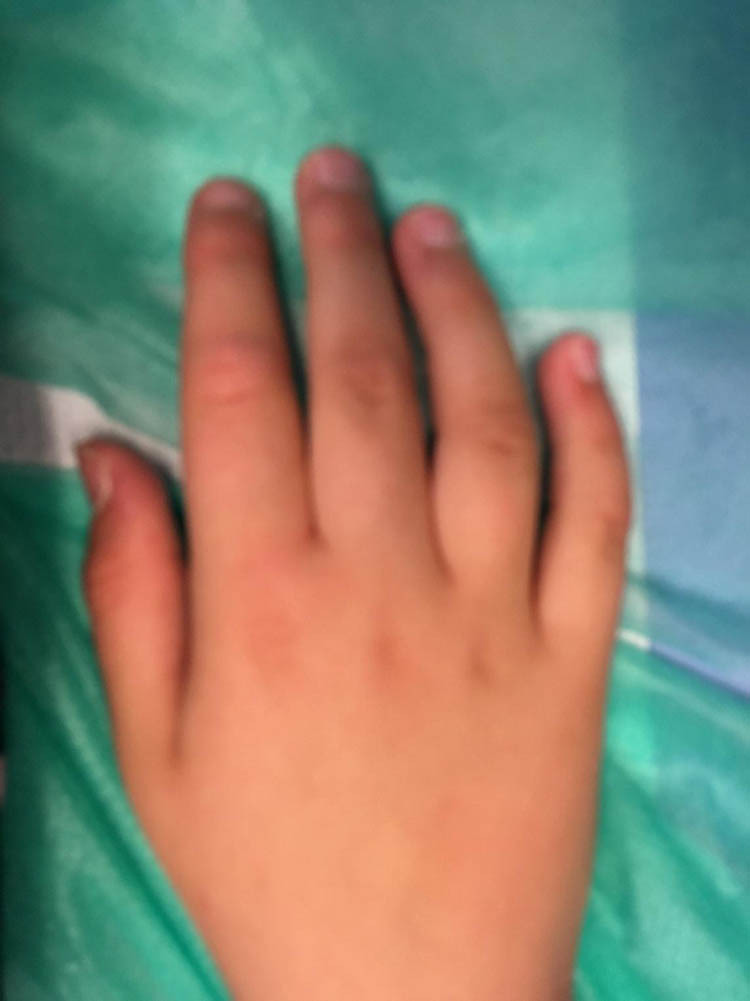
Small and short hands in girl with Cohen syndrome.

**Figure 4:**
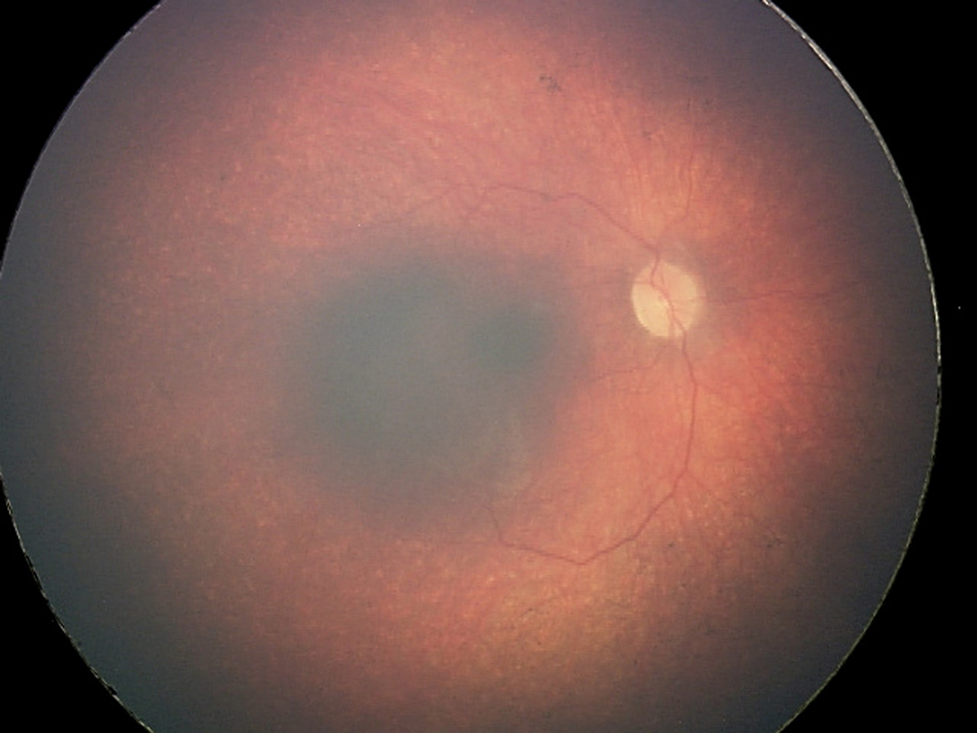
Right fundus photography of a girl with Cohen syndrome – note eyes pale optic discs and “salt and pepper” retinopathy.

**Figure 5:**
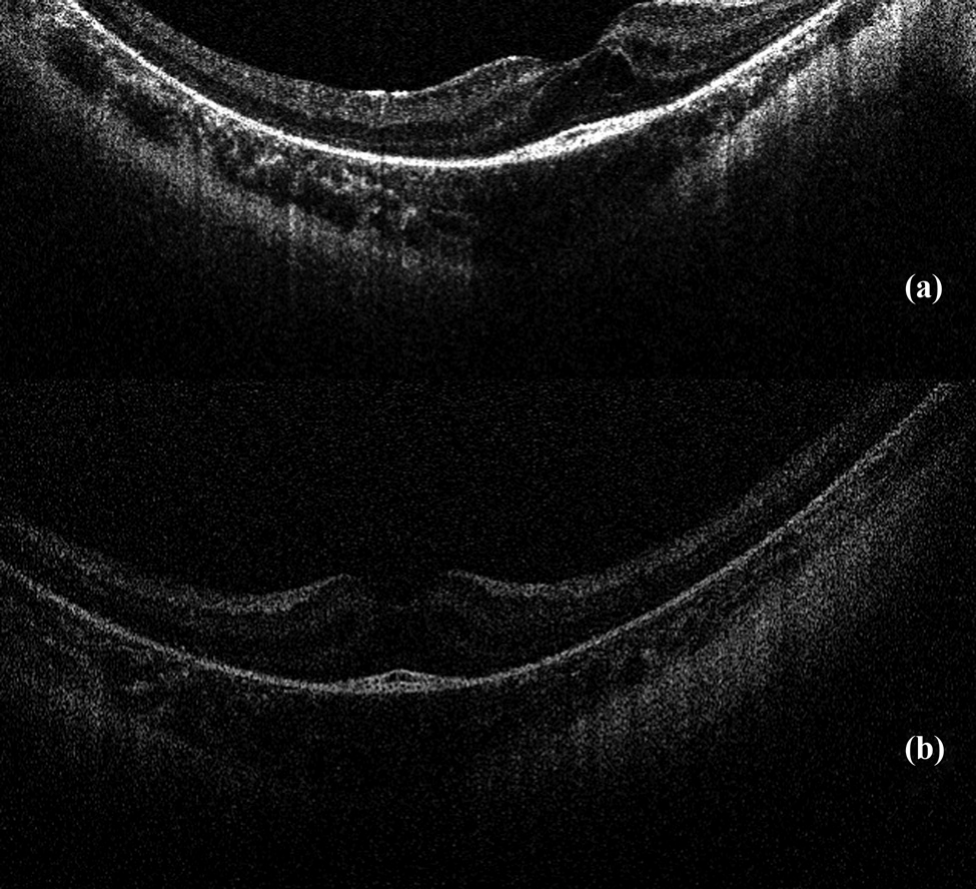
(a) and (b) Results of optical coherence tomography – macular edema in both eyes.

**Figure 6:**
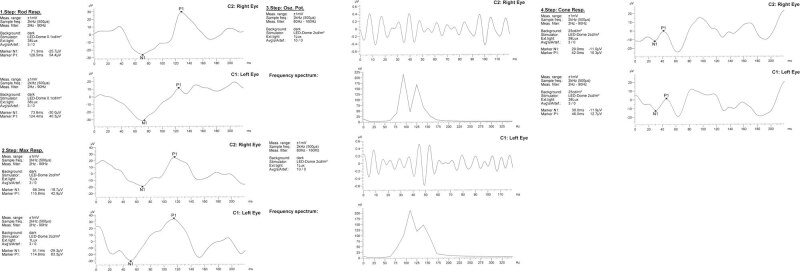
Electroretinography result.

The child was born by spontaneous delivery, at the 38th week of pregnancy with low birth weight −2,300 g. An echocardiographic examination was performed on the second day of life due to abnormal heart murmur. The examination showed atrial and ventricular septal defect and aortic coarctation. Some distinct features that draw attention to the girl are microcephaly, micrognation, facial dysmorphism: a prominent nasal bridge, thick hair and eyebrow, abnormalities of the palpebral fissures (downslanting and almond-shaped palpebral fissures) [Figure 2], prominent upper incisors and small and short hands [Figure 3]. During the first weeks of life, the patient developed larynx flaccidity and chronic neutropenia causing compromised immunity. At the ophthalmology department, the girl underwent a full ophthalmological examination, which was performed under short, inhalation anesthesia due to the patient’s failure of cooperation. Visual acuity was impossible to assess because of difficult contact with the child. Eyeball movement was normal, and no nystagmus was found. Refraction after accommodation paralysis revealed high myopic astigmatism: right eye −0.5 Dsph to −6.25 Dcyl ax 178 and left eye −3.25 Dsph to −4.5 Dcyl ax 180. The anterior segment was within normal limits in the slit lamp examination. Ophthalmoscopic examination of both eyes demonstrated pale optic discs and “salt and pepper” retinopathy, accompanied by arterial stenosis [Figure 4]. The intraocular pressure in the right eye was 12 mm Hg and in the left was 10 mm Hg. The axial length of both eyeballs was similar, i.e., in the right eye was 20.63 mm and in the left was 19.40 mm. The central thickness of cornea in the right eye was 413 µm and in the left was 439 µm. Handheld optical coherence tomography revealed macular edema in both eyes [Figure 5]. Electroretinography showed a reduced response amplitude of cones and rods [Figure 6].

## 3 Discussion

In Cohen syndrome, myopia is refractive in type due to high corneal and lenticular power, not the axial length of eyeball. This is most likely due to dysgenesis, corneal and ciliary body atrophy [1,4,10]. Kivitie-Kallio et al. [6] analyzed 22 patients with Cohen syndrome and reported high myopia and large astigmatism in the 0.5–6.0 Dcyl range in all the subjects.

Chandler et al. [11] examined 22 patients with genetically confirmed disease and found a refractive error in the range from −0.25 to −18 Dsph. Myopia was documented in 68% of patients before 5 years of age [11]. The authors agree that myopia and astigmatism progress with the patient’s age [4,10,12]. Our patient was diagnosed with myopia as early as at 3 years of age, which was accompanied by high astigmatism and thinning of the central thickness of the cornea.

In the literature, the most commonly reported retinal lesions quintessential of Cohen syndrome are bull’s eye maculopathy, chorioretinopathy, dystrophy with pigment granularity and “salt and pepper” retinopathy with narrowed vessels. At an advanced stage, retinopathy with bone spicule formed a classic picture of retinitis pigmentosa.

Kivitie-Kallio et al. [6] in a study of 22 subjects, the majority observed typical lesions for retinal retinopathy accompanied by pale optic disc in all the patients. Taban et al. [5] based on their own analysis detected pigment granularity and pale optic disc in all the patients. Chandler et al. [11] in 11 of 22 examined patients observed advanced, severe retinopathy with narrow vessels, bone spicule and pale optic disc.

Our patient is noticed by the pale, atrophic optic disc, which is accompanied by lesions in the retina, such as pigment granularity and narrow arterial vessels, while no typical bone spicule are found. Severe retinopathy with bone spicule has been reported in older people, so in our 6-year-old patient it may not be present yet.

Regarding lesions located in the macula, Beck et al. [9] presented a case of nonleaking cystoid macular edema in an 11-year-old patient with Cohen syndrome. Uyhazi et al. [3] focused on the analysis of the initial modification that occurs in the structure of the retina in the course of this syndrome. The authors described the case of a 13-month-old girl in whom OCT revealed loss of the interdigitation signal between the photoreceptor outer segments and the apical retinal pigment epithelium. Loss of only the photoreceptor outer segments was also noted, suggesting that these are the first visible symptoms of retina that later lead to macular edema and retinal dystrophy characteristic of Cohen syndrome.

Mutation of the *VPS13B* gene causes an incorrect function of the Golgi apparatus membrane protein, which affects, among others, the function of retinal photoreceptors [3,9,13]. It is assumed that the edema of the macula does not occur due to fluid accumulation, or an increase in vascular permeability, but only because of the impaired adhesion and splitting of several retinal layers [3,9,13]. The macular edema in hereditary retinal dystrophies is caused by a similar mechanism resulting from mutations in individual genes [13].

Bilateral macular edema was confirmed in the patient in handheld OCT. Macular lesions in Cohen syndrome have been reported in the literature, but only in two cases, a characteristic picture of OCT macular edema has been documented [3,9]. It can be assumed that this was due to the lack of access to this study technique in the past.

It cannot be excluded that the coexistence of the described ophthalmologic lesions may result from a homozygous deletion in our patient. Hennies et al. [4] examined the inheritance of Cohen syndrome in 20 patients. The authors confirmed the homozygous mutation in seven patients from the consanguineous parents and two patients from parents without known consanguinity – both from Poland. Heterozygous mutation has been documented in all other patients from unrelated parents.

## 4 Conclusion

It can be assumed that the type of mutation may affect the severity and diversity of ophthalmic features in Cohen syndrome. In a child with coexistence of high myopia and astigmatism, retinal dystrophy, pale optic disc and other abnormalities and facial dysmorphics, ophthalmologists should consider Cohen syndrome. Finding characteristic lesions in the eye in a group of children with suspected Cohen syndrome has a significant impact on making the correct diagnosis. Early diagnosis gives the possibility of appropriate visual rehabilitation and is crucial in the further development of the child.
